# Quantification and Tissue Localization of Selenium in Rice (*Oryza sativa* L., Poaceae) Grains: A Perspective of Agronomic Biofortification

**DOI:** 10.3390/plants9121670

**Published:** 2020-11-28

**Authors:** Ana Coelho Marques, Fernando C. Lidon, Ana Rita F. Coelho, Cláudia Campos Pessoa, Inês Carmo Luís, Paula Scotti-Campos, Manuela Simões, Ana Sofia Almeida, Paulo Legoinha, Maria Fernanda Pessoa, Carlos Galhano, Mauro A. M. Guerra, Roberta G. Leitão, José C. Ramalho, José Manuel N. Semedo, Ana Bagulho, José Moreira, Ana Paula Rodrigues, Paula Marques, Cátia Silva, Ana Ribeiro-Barros, Maria José Silva, Maria Manuela Silva, Karliana Oliveira, David Ferreira, Isabel P. Pais, Fernando Henrique Reboredo

**Affiliations:** 1 Earth Sciences Department, Faculdade de Ciências e Tecnologia, Universidade Nova de Lisboa, Campus da Caparica, 2829-516 Caparica, Portugal; fjl@fct.unl.pt (F.C.L.); arf.coelho@campus.fct.unl.pt (A.R.F.C.); c.pessoa@campus.fct.unl.pt (C.C.P.); idc.rodrigues@campus.fct.unl.pt (I.C.L.); mmsr@fct.unl.pt (M.S.); pal@fct.unl.pt (P.L.); mfgp@fct.unl.pt (M.F.P.); acag@fct.unl.pt (C.G.); djo.ferreira@campus.fct.unl.pt (D.F.); fhr@fct.unl.pt (F.H.R.); 2 GeoBioTec Research Center, Faculdade de Ciências e Tecnologia, Universidade Nova de Lisboa, Campus da Caparica, 2829-516 Caparica, Portugal; paula.scotti@iniav.pt (P.S.-C.); cochichor@mail.telapac.pt (J.C.R.); jose.semedo@iniav.pt (J.M.N.S.); ana.bagulho@iniav.pt (A.B.); jose.moreira@iniav.pt (J.M.); aribeiro@isa.ulisboa.pt (A.R.-B.); mjsilva@isa.ulisboa.pt (M.J.S.); abreusilva.manuela@gmail.com (M.M.S.); karliana.oliveira@ipbeja.pt (K.O.); isabel.pais@iniav.pt (I.P.P.); 3 Instituto Nacional de Investigação Agrária e Veterinária, I.P. (INIAV), Avenida da República, Quinta do Marquês, 2780-157 Oeiras, Portugal; sofia.almeida@iniav.pt; 4 LIBPhys, Physics Department, Faculdade de Ciências e Tecnologia, Universidade Nova de Lisboa, Campus da Caparica, 2829-516 Caparica, Portugal; mguerra@fct.unl.pt (M.A.M.G.); rg.leitao@fct.unl.pt (R.G.L.); 5 PlantStress & Biodiversity Lab, Centro de Estudos Florestais (CEF), Instituto Superior Agronomia (ISA), Universidade de Lisboa (ULisboa), Quinta do Marquês, Av. República, 1349-017 Lisboa, Portugal; anadr@isa.ulisboa.pt; 6 Instituto Nacional de Investigação Agrária e Veterinária, I. P. (INIAV), Estrada de Gil Vaz 6, 7351-901 Elvas, Portugal; 7 Centro Operativo e Tecnológico do Arroz (COTARROZ), 2120-014 Salvaterra de Magos, Portugal; pmarques@cotarroz.pt (P.M.); catia.leonardo.silva@gmail.com (C.S.); 8 ESEAG-COFAC, Avenida do Campo Grande 376, 1749-024 Lisboa, Portugal; 9 Instituto Politécnico de Beja (IPBeja), 7800-295 Beja, Portugal

**Keywords:** rice genotype, selenate, selenite, selenium biofortification

## Abstract

In worldwide production, rice is the second-most-grown crop. It is considered a staple food for many populations and, if naturally enriched in Se, has a huge potential to reduce nutrient deficiencies in foodstuff for human consumption. This study aimed to develop an agronomic itinerary for Se biofortification of *Oryza sativa* L. (Poaceae) and assess potential physicochemical deviations. Trials were implemented in rice paddy field with known soil and water characteristics and two genotypes resulting from genetic breeding (OP1505 and OP1509) were selected for evaluation. Plants were sprayed at booting, anthesis and milky grain phases with two different foliar fertilizers (sodium selenate and sodium selenite) at different concentrations (25, 50, 75 and 100 g Se·ha^−1^). After grain harvesting, the application of selenate showed 4.9–7.1 fold increases, whereas selenite increased 5.9–8.4-fold in OP1509 and OP1505, respectively. In brown grain, it was found that in the highest treatment selenate or selenite triggered much higher Se accumulation in OP1505 relatively to OP1509, and that no relevant variation was found with selenate or selenite spraying in each genotype. Total protein increased exponentially in OP1505 genotype when selenite was applied, and higher dosage of Se also increased grain weight and total protein content. It was concluded that, through agronomic biofortification, rice grain can be enriched with Se without impairing its quality, thus highlighting its value in general for the industry and consumers with special needs.

## 1. Introduction

Selenium is an essential micronutrient for human growth, acting in the antioxidant defense metabolism [1]. Yet, its deficiency is currently considered a public health problem that affects about 15% of the world’s population of six billion, with babies being the most vulnerable [2]. This physiological stress increases the risk of male infertility, heart disease, thyroid disorder, weakened immunity, cancer, and various inflammatory disorders [3]. Selenium deficiency is further associated with increased susceptibility to infections such as COVID-19 and HIV [4]. To address this physiological deficiency, the World Health Organization (WHO) recommends a daily intake of 30-40 µg Se for adults (with toxicity limits being reached if 400 µg are exceeded) while the National Academy of Sciences recommends 40–70 µg for male and 45–55 µg for female [5]. 

It is well known that in plants Se is directly related to soil bioavailability [6]. However, most soils in humid climates, temperate zones, and derived from sedimentary rocks have low levels of Se, being insufficient to produce food with high Se content [7]. To surpass Se deficiency fertilizers must be applied [8,9], as it increases plant production. Plant roots mobilize Se in the form of selenate (SeO_4_^2−^) and selenite (SeO_3_^2−^), but it was also found as complexes with organic matter or organo-mineral colloids. In roots, Se is transported by phosphate conveyors and quickly converted to organic forms such as selenocysteine (SeCys) and selenomethionine (SeMet) [10]. Selenate is translocated through the plasma membrane of root cells due to its high affinity with the sulphate carriers, therefore deposited in the xylem and translocated in the plant [10]. Once there, SeMet compounds are assimilated and redistributed, in the same way as sulphate [10].

The amount of micronutrients (namely Se) are low in staple foods, especially rice, which favors the development of deficiencies in about half of the world population [11]. Yet, to treat malnutrition, agronomic biofortification itineraries can be adopted as a strategy to increase mineral content of rice grains [12]. Through agronomic biofortification, Se concentrations in rice grains can be augmented when foliar fertilizers such as sodium selenate and sodium selenite are applied [8,9]. These chemical forms of Se are the main sources absorbed by this plant species and foliar fertilization is one of the most effective and safe approaches, because the applied product enters the plant through the cuticle or via stomata. Studies also pointed that biofortification is probably the most successful example of agronomic intervention [13], because selenate is very mobile, easily absorbed by the plants and accumulates in the grains with the form of SeMet and SeCys. In rice, Se also is a growth stimulator [14]. According to [15], Se further regulates the water potential in plants and shields plants against oxidative effects caused by internal and external stress [14]. Additionally, several studies point out that in rice foliar application of selenite is more effective than selenate [16]. 

A global survey of rice grains (*Oryza sativa* L., Poaceae) showed that 75% of the grains had an insufficient concentration of Se for human requirements [17]. Additionally, rice is one of the most socially and economically important cereals in the world [1]. For example, Bangladesh population consume around 400 g of rice per day, or around 145 kg per year [18]. In Portugal, the estimated annual consumption of milled rice is around 180,000 ton [19], with an average value of about 15 kg per consumer [20]. Accordingly, considering the growing importance of rice for human consumption, this work aimed to develop an itinerary of agronomic biofortification of rice grains (by foliar application of sodium selenate and sodium selenite) and to assess and locate the potential of Se increase in the grains of two rice genotypes.

## 2. Materials and Methods 

### 2.1. Experimental Fields

The trial was conducted, under field conditions, at the experimental station of the Rice Technological Center (COTARROZ), located in the middle of the lezíria ribatejana—Portugal (39°02′21.8″ N; 8°44′22.8″ W). Two new advanced rice lines of the breeding program carried out by the Instituto Nacional de Investigação Agrária e Veterinária (INIAV, Elvas, Portugal) were used. Those are denoted by codes OP1505 and OP1509. Trial duration occurred from 30 May to 2 November of 2018. During that period, the weather conditions associated with rice production were recorded by the weather station, located in Vila Franca de Xira (38.957° N, 8.988° E). In both varieties, analyses occurred in the paddy, brown, and white rice grains, and whole and refined flours.

Biofortification was carried out by foliar application with solutions of sodium selenate (Na_2_SeO_4_) and sodium selenite (Na_2_SeO_3_) with three replicates per genotype. Genotypes were sown in six row plots and then immediately irrigated. The experimental design was performed in randomized blocks and a factorial arrangement (5 concentrations × 2 forms selenium × 2 genotypes × 4 replicates = 80 plots). The plot size for each replication was 9.6 m^2^ (8 m length × 1.2 m width). The agronomic management of trials, namely the application of nitrogen fertilizers, control of weeds, insect pests, and diseases, and the water management (irrigation) were recommended and typically used for rice crops. The agronomic Se biofortification comprised three distinct phases. First Se application occurred at the end of booting, the second at anthesis and the third at the milky grain stage. Plants were sprayed with Na_2_SeO_4_ and Na_2_SeO_3_ using the following concentrations: 25, 50, 75, and 100 g Se·ha^−1^. Control plants were not sprayed at any time with Na_2_SeO_4_ or Na_2_SeO_3_. Grain harvest occurred at 02/11/2018 for both genotypes.

### 2.2. Climate, Soil, and Irrigation Water Analysis

During the rice growing cycle, from 30 May to 2 November 2018, air temperatures reached an average daily of 29 °C and 18 °C (with maximum and minimum values of 47 °C and 9 °C, respectively). The average rainfall was 0.67 mm, with a daily maximum of 17 mm and an accumulation of 0.7 mm. Quantification of soil organic matter considered 16 samples of about 100 g, using a rectangular grid of 5.70 × 4 m. Samples were collected from the rice paddy field, from surface to a 30 cm deep, being sieved (2.0 mm mesh) to remove stones, coarse materials and other debris. Soil weight was recorded, and after drying, at 105 °C, for 24 h (followed by 1 h desiccation), dry mass and percentage of moisture were determined. Samples were then heated, to 550 °C, for 4 h (i.e., until constant weight). Soil samples were removed from the muffle at 100 °C and desiccated until room temperature (*c.a.* 1 h). Samples weight was recorded to determine the percentage of organic matter. Following [21], pH and electrical conductivity of soil samples were measured, using a potentiometer. Briefly, after mixing, at a ratio of 1: 2.5 (g _soil_ mL^−1^_water milli-q_), for 1 hour with stirring (at 25 °C, for 30 min) in a thermal bath, determinations were carried out after decantation of the supernatant. Additionally, minerals content was analyzed, using a XRF analyzer (model XL3t 950 He GOLDD+) under helium atmosphere [22].

Water quality considered physical (pH, temperature, and electrical conductivity) and chemical (bicarbonate, sulfate, chloride, sodium, calcium, magnesium, potassium, nitrate, and phosphate) parameters. Electrical conductivity (EC) and pH were determined using a Consort multiparameter analyzer (C 6030) and SP21 (pH) and SK20T (CE) electrodes. Calcium, Na, K, and Mg ions were quantified using a Metrohm (Model 761 Compact IC) chromatograph, equipped with column and pre-column (Metrosep cation 1-2, 6.1010.000), using an eluent mixture (4mM tartaric acid/1 mM dipicolinic acid) at a flow rate of 1.00 mL/min and a sample injection of 10.0 μL. Alkalinity/bicarbonate was determined by titration, in 100 mL of water samples, using 0.1 N hydrochloric acid as titrant, in the presence of 0.1% methyl orange [23]. Chloride, sulphate, nitrate, and phosphate ions were quantified by photometry (Spectroquant NOVA 60, Merck), using specific kits (1.14897, 1.14779, 1.14773, and 1.14842). Water classification in the soils of the rice paddy field, considering dominant ions, followed Piper (1944). Sodium adsorption index was determined and related to the electrical conductivity, in classes C and S. The Langelier saturation index was also estimated from the pHe (equilibrium pH), at a reference temperature a 20 °C, to determine the fouling or aggressiveness of the water relatively to calcium carbonate.

### 2.3. Analysis of Grain Selenium Content and Tissues Location

The quantification and localization of Se in tissues was determined in harvested grains from control and sprayed plots with selenate and selenite at 0 and 100g Se·ha^−1^ using the µ-EDXRF system (M4 Tornado™, Bruker, Karlsruhe, Germany) [24]. The X-ray generator was operated at 50 kV and 100 µA without the use of filters, to enhance the ionization of low-Z elements. To a better quantification of Se, a set of filters between the X-ray tube and the sample, composed of three foils of Al/Ti/Cu (with a thickness of 100/50/25 µm, respectively) was used. All the measurements were performed with 600 µA current. The values of the content of Ca were obtained through the average of four readings taken by the device. Detection of fluorescence radiation was performed by an energy-dispersive silicon drift detector, XFlash™, with 30 mm^2^ sensitive area and energy resolution of 142 eV for Mn Kα. To better evaluate the distribution mapping of Se, rice grain was cut, at the equatorial region, into slices with a stainless steel surgical blade. Measurements were carried out under 20 mbar vacuum conditions and performed directly on the two sides of grains, first in the mapping mode, and then with point analysis on interest sites. These point spectra were acquired during 200 s. 

### 2.4. Thousand Grain Weight, Colorimetry and Crude Protein Content Analysis

For each genotype and treatment, 1000 grains were picked randomly and weighed in triplicate. Subsequently, grains were hulled and whitened, as described in [9].

Determination of the colorimetric parameters of grain samples, using a fixed wavelength, followed [25]. Grains colorimetric parameters, after harvesting, were determined with a scanning spectrophotometric colorimeter (Agrosta, European Union). The sensor provides a 40 nm full-width half-max detection and covered the entire visible area. This sensor has 6 phototransistors, each with sensitivity in a specific spectrum, at 380 (violet), 450 (blue), 500 (green), 570 (yellow), 600 (orange), and 670 (red) nm. Light was provided with a white LED having a wide spectrum across the visible area. The measurements were carried out in quadruplicates on harvested rice grains.

Crude protein was determined after quantification of total nitrogen, using the Kjeldhal method [26], and converting this value into crude protein considering that total nitrogen is in the protein form. 

### 2.5. Statistical Analysis

Data were statistically analyzed using a One-Way or Two-Way ANOVA (*p* ≤ 0.05), to assess differences among treatments. Based on the results, a Tukey’s for mean comparison was performed, considering a 95% confidence level. For the Two-Way ANOVA, different letters in the columns (a, b, c, d) and lines (r, s) indicate significant differences among the different treatments of each genotype, or between the same treatments for the different genotype, respectively. Statistical analysis was performed with an IBM SPSS Statistics 20 program. 

## 3. Results

### 3.1. Characterization of Rice Field and Yield

Rice paddy soils was found to be suitable for crops management (slightly acid, in the pH range 5.5–6.5; with soil conductivity of 0.223 dS/m). Soil analysis also demonstrated relevant contents of Fe, Ca, K, S, P, and Mg, and high contents of Pb, As, whereas Se remained lower than 1 ppm (Table 1).

Relatively to water irrigation (Table 1) of the rice paddy field, it was found to be of underground origin. Water collected in the deep hole revealed an intermediate mineralization (concentration of salts evaluated, in terms of electrical conductivity, between 0.250–0.750 dS/m, at 20 °C) and facies sodium bicarbonate chloride; belong to class C2S1, with a sodium adsorption index of 1.98. This water was also found to be under saturated with calcium carbonate, being very corrosive, with pH 8.4 and Langelier saturation index of −1.8. From the capture to the field, irrigation water evolved, being enriched in Ca, Mg, and Na cations, losing K, decreasing HCO_3_ and increasing in Cl. Levels of Se remained lower than 1 ppm. 

Inundated water in paddy field showed increases on mineralization and electrical conductivity from 0.376 dS/m to 0.800 dS/m, being the rise more significant in Na and Ca ions. Climate during rice crop season was characterized by maximum and minimum average temperatures of 29 °C and 18 °C (with absolute maximum and minimum values of 47 °C and 9 °C, respectively), with a daily average precipitation of 0.67 mm and a daily maximum of 17 mm.

Under the defined rice growth conditions, it was found that, at harvest, the average yields (in kg ha^−1^) were for OP1505, 4669 and 4917 and for OP1509, 5568 and 4870 (for both genotypes, after application of selenate and selenite, respectively)

### 3.2. Selenium Accumulation in Rice Grains

Selenium accumulation in brown rice grains of OP1505 and OP1509, between the control and the highest treatment, increased in both selenate and selenite treatments (Table 2).

**Figure 1:**
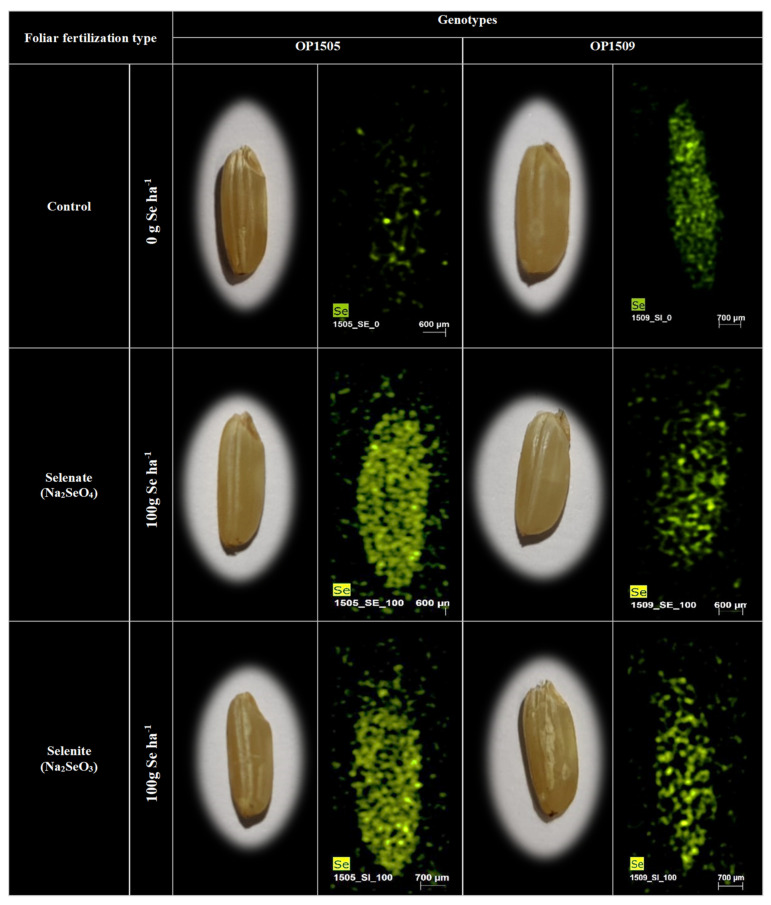
Location of Se in rice grains of OP1505 and OP1509 in the control and after foliar fertilization with sodium selenite and with sodium selenate at 100 g Se·ha^−1^.

In OP1505, selenate spraying resulted in significantly higher Se contents relatively to the control, except in treatment 25 g Se·ha^−1^ (Table 2). Therefore, under the defined experimental conditions, it was found that 25 g Se·ha^−1^ was not sufficient to promote Se biofortification in rice. Moreover, natural fortification with Se can already be induced to some extend with 50, 75, and 100 g Se·ha^−1^ (i.e., the contents increased from 4.75 to 17.17 ppm) resulting in a 7.1 fold increase. Selenium levels increased with the augmentation of selenite concentration, from 2.01 ppm (control) up to 16.89 ppm (except for treatment 75 g Se·ha^−1^). In OP1509, selenate triggered a 4.9 fold increase of Se in the grain until the last treatment, from 1.37 to 6.72 ppm. When selenite was applied, the levels of Se were significantly higher than the control, increasing from 2.20 ppm to 13.1 ppm until treatment 50 g Se·ha^−1^. Comparing data of Se accumulation from both genotypes, selenite treatments were reached values of 8.4 and 5.9 fold in genotypes OP1505 and OP1509, respectively. In both genotypes, with selenate or selenite application, Se were evenly distributed in the grain (Figure 1).

### 3.3. Grain Weight, Colorimetry, and Crude Protein Content Analysis

Foliar fertilization with Se, in both forms, did not significantly affected grain weight in OP1505 and OP1509 (Table 3), yet grain weight remained higher in OP1509, being these results in accordance with specific genetic features of each genotype.

**Figure 2:**
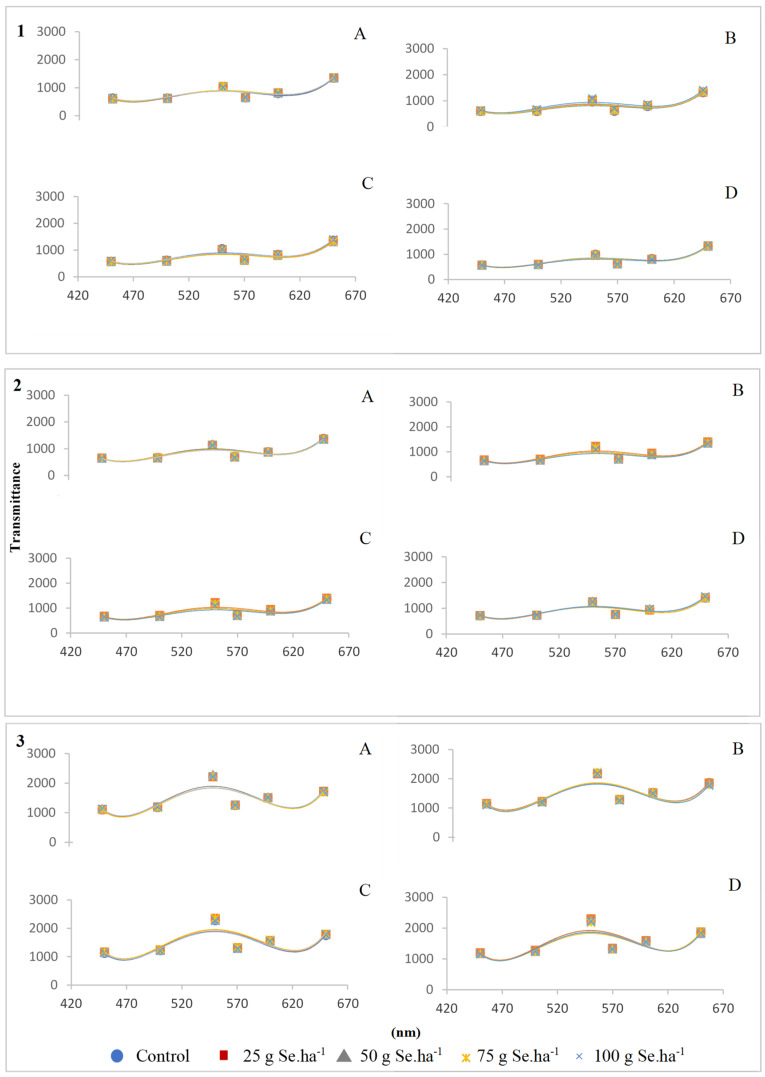
Average colorimetric transmittance (*n* = 4), in the 450–650 nm band, of post-harvested paddy (**1**), brown (**2**) and white (**3**) grains, of OP1505 (**A**—selenate; **B**—selenite) and OP1509 (**C**—selenate; **D**—selenite).

After application of selenate or selenite, colorimetric analysis of paddy, brown, and white grains from both genotypes, showed (Figure 2) two peaks (at 550 and 650 nm), corresponding to green to yellow and yellow to red transitions. It was found that peaks became more accentuated after industrial processing of rice grains but, in general, among treatments color parameters remained similar.

Genotype OP1505 treated with selenate showed, in whole flour, maximum protein values with treatments 50 and 100 g Se·ha^−1^, but significant differences were not found (Table 4). With selenite spraying, flour of OP1505 showed, relatively to the control, a slight increase between 25–75 g Se·ha^−1^, but again significant differences could not be detected (Table 4). The control of whole flour of OP1509 showed higher values when both Se forms were applied (except for the application of 50 g Se·ha^−1^, which registered the maximum protein content, 6.49% with selenite and 7.15% with selenate) yet, again, significant differences could not be detected. 

Refined flour of OP1505, with selenate spraying at a concentration of 25 and 100 g Se·ha^−1^, showed the lowest contents of grain protein. A different trend was observed when selenite was applied in this genotype, with grain protein increasing from 4.63% (control) to 5.23% (with spraying with 100 g Se·ha^−1^). Nevertheless, significant differences also could not be found (Table 4). In refined flour of OP1509, the contents of protein remained higher in the control after spraying with both forms of Se (Table 4) but, still no significant differences were detected (Table 4). 

## 4. Discussion

Accumulation of Se in plant tissues is closely related with its availability in soils, which implicates pedology genesis [17]. Yet, cultivation conditions also has significant influence in Se uptake by plants [27]. In this context, this study also considered that the development of the life cycle of rice is closely linked to soil characteristics, electrical conductivity, contents of organic and mineral matter (Table 1) and irrigation (as water quality can strongly decrease plant growth and yield and Se application can improve water efficiency by rice) [6]. Besides, weather conditions were also considered, as the interactions between genotypes and environment can be a determinant to achieve maximum productivity. Nevertheless, it is fundamental to develop new strategies to increase Se content in cereals, namely by fertilization and to cultivate varieties with higher accumulation potential [28]. In this context, considering that soil and irrigation water properties were adequate for rice production, with the goal of increasing Se content in rice grain, genetic breeding and agronomic biofortification were combined, testing different spray concentrations of Se, from two different chemical sources, with two different genetic backgrounds. However, although foliar application of Se triggers direct transfer and accumulation in plants, followed by diffusion of Se ions in the leaf epidermis, if the threshold of toxicity is surpassed, proliferation of reactive species of oxygen (ROS) induce high lipid peroxidation rates in cell membranes [29].

It is well known [1] that increasing contents of Se in rice grains depends on the concentration and form of Se applied as well as on the characteristics of the genotypes. Our study showed that for both genotypes, both forms of the Se applied promoted biofortification. (Table 2). This natural fortification eventually is a consequence of longer periods in flooded conditions [30], which can result in higher concentrations of total and extractable Se. That flooding might lead to reductive dissolution of iron oxyhydroxides, which are important sorbents of Se forms like selenite [10]. In brown grain of OP1505, there was a decrease in grain Se accumulated as doses of selenite were increased, which suggests inhibitory interactions between the Se form applied and the plant, as Se can interfere with enzymatic processes and antioxidant metabolism [29]. Through tissue location data, it was possible to verify that, regardless of the form applied, Se was evenly distributed in the grain (Figure 1), as most of the micronutrients [13,31]. This aspect became particularly relevant since the paddy and brown grains must be processed to obtain the white rice grains required for human consumers. Accordingly, the white rice grains of OP1505 might keep the high Se content after industrial processing (i.e., dehusking, whitening, and milling) [8], since upon biofortification Se is spread in all the white rice (Figure 1; Table 2). 

The highest grain weight was verified in the brown and white grain of OP1509 (Table 3), although there were no significant changes, which further indicated that the applied itinerary of biofortification did not affected productivity since the threshold of toxicity was not reached. Similarly, the absence of substantial variations of color parameters of paddy, brown, and white rice grains among treatments (Figure 2), further pointed that toxicity was not reached until harvesting. The average of total protein contents in the flours ranged between 5.8–7.2% and 4.6–5.8% in the whole flour and refined flour, respectively (Table 4), further indicating that Se kept the nutritional value, largely due to the interaction effects between Se and N [31,32]. Indeed, high content of Se interferes with the sulfur metabolism, which can affect the N metabolism and yield [33]. Additionally, the higher protein level in the whole flour of OP1505 with the application of selenite, suggested a greater amount of amino acid, that can additionally increase Maillard reactions generating brown compounds, which might contribute to surface coloring of cookies [34]. Yet, although there is a positive impact regarding the color in consumer’s perception, high protein contents can interfere with the viscosity of the rice flour pasta [35,36]. Flours with lower protein content will have greater applicability in products that aim higher pasta viscosity. 

## 5. Conclusions

*O. sativa* sprayed with selenate or selenite concentrations ranging between 25–100 g Se·ha^−1^ did not surpass the threshold of toxicity. At different extends Se remained in the grain tissues regardless of the form applied. This study addressed a higher agronomic biofortification for OP1505, with the application of both Se forms allowing a greater increase of Se in rice grains. Grain yield remained higher with selenite in OP1505 and selenate for OP1509, yet grain weight did not vary significantly in both genotypes. Besides, total protein, although showing some non-significant variation among treatments, in each genotype did not revealed relevant changes (in whole and refined flour) through application of both chemical forms of Se. Accordingly, the developed itinerary for Se biofortification can be used as an agronomic strategy to increase public health, without affecting rice nutrition or protein levels. 

## Figures and Tables

**Table 1:** Soil analysis (at 0–30 cm deep) and water irrigation (samples collected at points: 1—irrigation hole; 2—field entrance; and 3—inside the field trial) analysis of rice paddy field trial.

**A**											
**Soil Analysis (0–30 cm Deep)**											
**pH**	**Electrical Conductivity**	**Organic Matter**	**Ca**	**K**	**P**	**Fe**	**S**	**Mg**	**Pb**	**As**	**Se**
	dS/m	%					ppm				
5.86	0.223	1.32	0.16 ± 0.01	2.48 ± 0.04	1.50 ± 0.06	0.28 ± 0.01	299 ± 114	682 ± 99	11.6 ± 0.61	13.1 ± 0.18	1<
**B**											
**Water Analysis**											
	**pH**	**Electrical Conductivity**	**Ca^2+^**	**K^+^**	**Mg^2+^**	**Na^+^**	**Cl^−^**	**HCO_3_^−^**	**SO_4_^2−^**	**PO_4_^3−^**	
		dS/m	Mg.l^−1^ (meq.l^−1^)								
**1**	6.9	0.376	9.4 (0.5)	8.1 (0.2)	4.5 (0.4)	30.2 (1.3)	52 (1.4)	67.1 (1.1)	45 (0.9)	<1.5 (<0.04)	
**2**	6.3	0.420	13.2 (0.7)	2.3 (0.06)	5.2 (0.4)	39.1 (1.7)	76 (2.1)	54.9 (0.9)	43 (0.8)	<1.5 (<0.04)	
**3**	6.6	0.800	27.6 (1.4)	14.4 (0.4)	7.1 (0.6)	62.5 (2.7)	131 (3.7)	85.4 (1.4)	74 (1.5)	<1.5 (<0.04)	

**Table 2:** Average (ppm) ± SE (*n* = 4) of Se (considering dry weight as a reference) in the brown grains of *Oryza sativa* L. (Poaceae), genotypes OP1505 and OP1509 at harvesting. Letters a, b, c, d indicate significant differences among treatments of each genotype and letter r, s between both genotypes (*p* ≤ 0.05).

Treatments (g Se·ha^−1^)	OP1505	OP1509
	**Na_2_SeO_4_**	
**Control**	2.43 ± 0.12as	1.37 ± 0.07ar
**25**	2.13 ± 0.11ar	2.47 ± 0.12br
**50**	6.98 ± 0.35cr	6.72 ± 0.34cr
**75**	4.75 ± 0.24br	6.60 ± 0.33cr
**100**	17.2 ± 0.86ds	6.45 ± 0.32cr
	**Na_2_SeO_3_**	
**Control**	2.01 ± 0.10ar	2.20 ± 0.11ar
**25**	5.19 ± 0.26cr	7.34 ± 0.37cs
**50**	6.12 ± 0.31cr	13.1 ± 0.65ds
**75**	3.51 ± 0.18br	5.32 ± 0.27br
**100**	16.9 ± 0.84ds	8.15 ± 0.41cr

**Table 3:** Average (g) ± (*n* = 4) of 1000-grain weight of rice submitted to foliar application with sodium selenate and sodium selenite. Letter a revealed the absence of significant differences among treatments of each genotype (*p* ≤ 0.05).

Treatments (g Se·ha^−1^)	Paddy		Brown Rice		White Rice	
	OP1505	OP1509	OP1505	OP1509	OP1505	OP1509
	**Na_2_SeO_4_**					
**Control**	26.7 ± 0.47a	31.6 ± 0.67a	24.2 ± 0.41a	27.0 ± 0.36a	21.7 ± 0.16a	23.6 ± 0.19a
**25**	27.2 ± 0.25a	31.5 ± 0.61a	24.1 ± 0.34a	27.3 ± 0.57a	20.9 ± 1.38a	23.4 ± 0.40a
**50**	28.5 ± 0.48a	31.6 ± 0.32a	24.0 ± 0.27a	26.9 ± 0.31a	21.4 ± 0.35a	23.8 ± 0.48a
**75**	27.6 ± 0.31a	30.8 ± 0.80a	24.4 ± 0.25a	27.2 ± 0.16a	20.9 ± 0.23a	23.7 ± 0.47a
**100**	27.7 ± 0.31a	32.2 ± 1.71a	24.3 ± 0.33a	27.6 ± 0.69a	21.5 ± 1.42a	23.9 ± 0.44a
	**Na_2_SeO_3_**					
**Control**	26.4 ± 1.19a	29.3 ± 0.52a	24.2 ± 0.38a	25.9 ± 0.63a	21.0 ± 0.27a	24.0 ± 0.81a
**25**	26.7 ± 0.07a	30.2 ± 0.51a	24.3 ± 0.19a	27.0 ± 0.27a	21.1 ± 0.39a	24.4 ± 0.14a
**50**	26.6 ± 0.68a	29.8 ± 0.67a	24.1 ± 0.11a	26.6 ± 0.17a	21.5 ± 0.24a	23.9 ± 0.31a
**75**	28.3 ± 0.51a	28.8 ± 1.01a	23.6 ± 0.25a	25.9 ± 0.38a	21.3 ± 0.18a	23.5 ± 0.37a
**100**	27.2 ± 0.62a	30.7 ± 1.38a	23.9 ± 0.20a	26.0 ± 0.38a	20.6 ± 0.12a	23.7 ± 0.17a

**Table 4:** Protein in whole and refined rice flours submitted to foliar application of sodium selenate and sodium selenite. Letter a revealed the absence of significant differences among treatments of each genotype and letter r between both genotypes (*p* ≤ 0.05).

Treatments (g Se·ha^−1^)	Whole Flour		Refined Flour	
	OP1505	OP1509	OP1505	OP1509
	(%)			
	**Na_2_SeO_4_**			
**Control**	6.39a,r	6.24a,r	5.53a,r	5.47a,r
**25**	6.05a,r	5.98a,r	5.16a,r	5.15a,r
**50**	6.62a,r	6.49a,r	5.83a,r	4.63a,r
**75**	5.82a,r	6.06a,r	5.40a,r	5.00a,r
**100**	6.62a,r	6.16a,r	5.24a,r	4.99a,r
	**Na_2_SeO_3_**			
**Control**	6.15a,r	6.70a,r	4.63a,r	5.65a,r
**25**	6.32a,r	5.83a,r	4.64a,r	4.75a,r
**50**	6.64a,r	7.15a,r	4.72a,r	5.32a,r
**75**	6.82a,r	6.65a,r	4.72a,r	5.46a,r
**100**	6.30a,r	6.64a,r	5.23a,r	4.98a,r
